# An empowerment intervention for Indigenous communities: an outcome assessment

**DOI:** 10.1186/s40359-015-0086-z

**Published:** 2015-08-21

**Authors:** Irina Kinchin, Susan Jacups, Komla Tsey, Katrina Lines

**Affiliations:** The Cairns Institute, James Cook University, PO Box 6811, Cairns, QLD 4870 Australia; College of Business, Law & Governance, James Cook University, PO Box 6811, Cairns, QLD 4870 Australia; Act for KIDS, Box 3387, South Brisbane, QLD 4101 Australia

**Keywords:** Empowerment, Intervention, Indigenous communities, Outcome assessment, Aboriginal

## Abstract

**Background:**

Empowerment programs have been shown to contribute to increased empowerment of individuals and build capacity within the community or workplace. To-date, the impact of empowerment programs has yet to be quantified in the published literature in this field. This study assessed the Indigenous-developed Family Wellbeing (FWB) program as an empowerment intervention for a child safety workforce in remote Indigenous communities by measuring effect sizes. The study also assessed the value of measurement tools for future impact evaluations.

**Methods:**

A three-day FWB workshop designed to promote empowerment and workplace engagement among child protection staff was held across five remote north Queensland Indigenous communities. The FWB assessment tool comprised a set of validated surveys including the Growth and Empowerment Measure (GEM), Australian Unity Wellbeing Index, Kessler psychological distress scale (K10) and Workforce engagement survey. The assessment was conducted pre-intervention and three months post-intervention.

**Results:**

The analysis of pre-and post-surveys revealed that the GEM appeared to be the most tangible measure for detecting positive changes in communication, conflict resolution, decision making and life skill development. The GEM indicated a 17 % positive change compared to 9 % for the Australian Unity Wellbeing Index, 5 % for the workforce engagement survey and less than 1 % for K10.

**Conclusions:**

This study extended qualitative research and identified the best measurement tool for detecting the outcomes of empowerment programs. The GEM was found the most sensitive and the most tangible measure that captures improvements in communication, conflict resolution, decision making and life skill development. The GEM and Australian Unity Wellbeing Index could be recommended as routine measures for empowerment programs assessment among similar remote area workforce.

## Background

Empowerment and community participation are major strategies used worldwide to increase social cohesion, which in-turn can be used as tools to reduce poverty (Tsey et al. 2009). When offered in workplace or community settings, empowerment programs have been shown to improve workforce retention, job satisfaction, performance, service delivery and concern for others (Fulford and Enz 1995). In community settings, they have been shown to improve individual self-worth, resilience, problem solving abilities, health and interpersonal communication, with a subsequent reduction in interpersonal violence (Haswell et al. 2010).

The common delivery method of empowerment programs involves a group setting as this allows participants to explore topics such as ‘beliefs and attitudes’, ‘conflict resolution’, ‘crises’ and ‘relationships’, etc., and to compare their views and behaviours to others. Through their participatory nature, empowerment programs can empower individuals to take responsibility for their everyday lives (Maton 2008, N. Wallerstein 2006, Zimmerman et al. 1992, Tsey and Every 2000, Whiteside et al. 2006, Tsey et al. 2010), and positively influence individuals to make better choices and modify their behaviours (N. Wallerstein 1992).

Often wider community benefits stem from small-scale empowerment programs (Tsey et al. 2007b). Wider community capacity is obtained when individual improvements snowball into improved family and social cohesion; this can further snowball into whole-of-community improvements (Israel et al. 1994, Tsey et al. 2005a). Building community wide capacity can enable wider issues to be addressed, such as poor school attendance, interpersonal violence, drug and alcohol misuse, chronic disease management and community crime rates (Tsey et al. 2007b). However, often these processes take many years to achieve change beyond the individual level (Tsey et al. 2009).

### Indigenous-developed Family Wellbeing (FWB) program

Australia’s Indigenous population have a well-documented history of alienation, and discrimination. A fundamental challenge lays in tackling the marginalised position of Indigenous people within Australian society. The FWB program was developed in the early 1990s by a group of Indigenous Australians to address socio-economic disadvantage and health inequality, in addition to grief, loss and stress, which are major components of everyday life in many Indigenous communities (Haswell et al. 2010). FWB covers issues of empowerment and wellbeing including but not limited to aspects of strengths, relationships, emotions and ways of dealing with crisis (Tsey et al. 2005b).

To increase capacity and strengthen social cohesion, empowerment programs have been offered in many Indigenous Australian communities, with substantial effects (Tsey et al. 2005a, 2007a, 2009). Many dramatic changes have been reported when empowerment programs were delivered in communities reporting high levels of interpersonal conflict and violence, unemployed or incarcerated people (Tsey and Every 2000, Whiteside et al. 2006). However, these published findings have drawn upon qualitative analysis. To-date, no empowerment programs have been quantified in the published literature in this field. The paper addresses this gap in the research. It seeks to quantify outcomes of the empowerment intervention and identify the best measurement tool for future impact evaluations.

## Methods

### Study population demographics

Upon invitation, an empowerment program was delivered to staff of a child protection agency by a research team consisting of academic staff from James Cook University and other partnering universities.

Out of the total 71 staff members, 5 were unable to attend the workshop. 66 employees formed a representative sample of the total population.

All participants (*n* = 66) were employees of the agency. 89.4 % (59) of the participants identified as Indigenous, 81.8 % (54) were female and all were aged 24–65 (missing in 2 participants), Table 1.

**Table 1:** Demographics, baseline study population characteristics

Baseline sample population characteristics	Number	Percent
Gender		
Male	8	12.1 %
Female	54	81.8 %
Indigenous identified	59	89.4 %
Age group		
<34	14	21.2 %
35 to 54	38	57.6 %
>55	13	18.2 %
Education		
<year 10	6	9.1 %
Year 10	25	39.4 %
Year 12	17	37.9 %
TAFE education	35	53.0 %
University education	7	10.6 %
Employment years		
<2 years	41	62.1 %
>3 years	23	34.9 %
Total baseline	66	100 %

### The empowerment intervention

FWB is a program delivered in workshops. FWB was delivered to the child protection workers from five remote Indigenous communities in six workshops over three days. One workshop for managers and coordinators was held in a regional centre in August 2012. A series of five workshops were run across the communities in locations where the child protection agency offer services from February 2013 to September 2013.

Workshops provided the foundational stage of the FWB program including topics of Group agreement, Human qualities, Basic human needs, Understanding relationships, Life journey, Conflict resolution, Understanding emotions and crisis, Life journey, Loss and grief and Beliefs and attitudes (Tsey and Every 2000).

The purpose of the empowerment intervention was to enhance workers’ self-esteem, interaction at the family and community levels, reduce social alienation and increase opportunities for self-development those social and emotional ‘blockages’ or barriers preventing many Indigenous Australians from achieving their life potential.

### Measurement tool

A questionnaire which comprised a set of validated surveys (*n* = 4) was administered at the beginning and three months after the FWB program delivery. The questionnaire was designed to measure empowerment and wellbeing as well as general workplace experiences and attitudes given that participants were involved in the empowerment program as an aspect of their work roles.

#### Measuring empowerment and wellbeing

One of the measures used was the validated Growth and Empowerment Measure (GEM) survey which comprised a 14-item Empowerment Scale. The GEM was developed as a tool to measure the process and outcomes of empowerment interventions such as FWB (Haswell et al. 2010). The GEM identifies emotional wellbeing and outcomes of empowering change important to Indigenous people (Haswell et al. 2010) at individual, family and organisational levels. The following domains of empowerment and wellbeing are examined by the GEM survey: self-capacity, inner peace, strength, happiness and connectedness.

Another survey tool used was the Australian Unity Wellbeing Index developed by researchers at Deakin University (Cummins and Schafer 2011). The index is based on a regular national population survey of 2000 participants and has been conducted since 2001. The dimensions covered by the Australian Unity Wellbeing Index in the measurement of wellbeing are: health; community; achievement in life; life as a whole; future security; spirituality; standard of living; relationships and safety.

A third survey tool used was the Kessler psychological distress scale (K10). The original K10 is a brief 10-item self-report survey designed to measure the level of distress and severity of psychological symptoms and is widely and commonly used as a clinical outcome measure (Brooks et al. 2006).

The GEM and the K10 used “Likert” scaling from one to five. The Australian Unity Wellbeing Index was scaled between zero and ten. Higher scores on the GEM and the Australian Unity Wellbeing Index indicated higher levels of empowerment and self-efficacy. Higher scores on the K10 indicated higher levels of anxiety.

#### Workforce engagement survey

The workplace questions were aligned to the specific circumstances of staff members. Standard workplace questions were sought that related to communication, attitudes toward management, perceived benefit of the participant’s work role to the community and job satisfaction. The Australian Public Service Indigenous employee survey (Australian Public Service Commission 2009) was reviewed as were some standard employee survey questions. This survey provided reference material for questions which were included to measure workforce engagement (McEwan et al. 2010).

### Data collection and statistical methods

The intervention targeted the entire workforce including managerial staff. Participants were requested to complete the questionnaire before and three months after completion of the three-day FWB workshop.

Not all participants completed the post-workshop survey (pre-survey *n* = 66, post-survey *n* = 50). Using a sample size calculator for 80 % power and alpha error of 0.05, assuming a t-distribution, for a difference in means of 0.4, this study would require 63 participants, assuming only 47 would be tested again in the follow up visit.

As data were de-identified, the collection of post-survey questionnaires could not be linked with pre-collected surveys, thus analysis conducted was ‘unmatched’. To compare pre- versus post-FWB responses, t-tests were performed  for each question. Then a linear regression was applied to the averaged individual responses for each set of questions to enable a larger scale comparison of the questionnaire function. This enabled the calculation of an overall effect size measure (pre versus post) for each questionnaire. This study followed the methodology of Berry et al. (2012) using *r* for effect size, with *r* greater than 0.5 considered large, greater than 0.3 considered medium and greater than 0.1 considered small. Figures included indicate the mean pre versus post FWB workshop effect with 95 % confidence intervals (CI) for clarity.

### Ethics

Ethics approval was obtained and procedures were followed in accordance with the standards of the James Cook University Ethics Committee (number H4719). A written informed consent was obtained from all participations. Children were not included in the study.

## Results

**Fig. 1:**
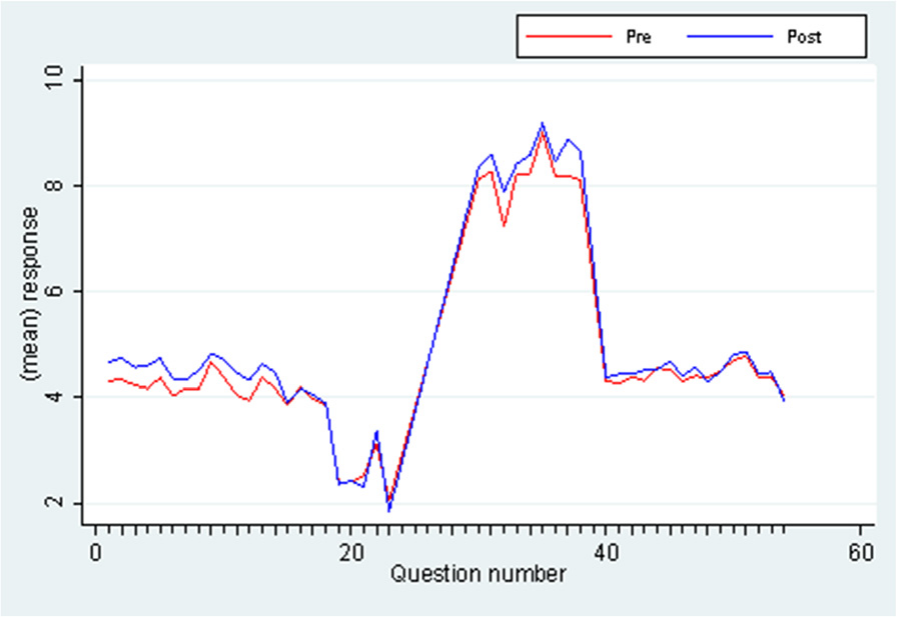
FWB program survey. Mean responses, pre and post

Analysis was conducted to examine effect sizes between pre- and post-surveys. Based on comparisons of participants’ mean responses before and after participation in the workshops, results indicated variation in the effect sizes for the four subscales of the FWB questionnaire (Table 2, Fig. 1) with stronger effect sizes indicated by larger gaps between pre versus post mean responses. The effect size for the GEM was highly sensitive to changes in the current FWB program population. The effect size for the K10 was small and not significant. The effect size for Australian Unity Wellbeing Index was double the effect size of the Workforce engagement survey, Table 2.

**Table 2:** Survey comparison

Survey name	Effect size (r)	*P* value
GEM (Q1-14)	17 %	<0.001
K10 (Q19-23)	1 %	0.715
Australian Unity Wellbeing Index (Q30-38)	9 %	0.004
Workforce engagement survey (Q40-54)	5 %	0.060

The GEM responses on self-capacity, inner peace, strength, happiness and connectedness (questions 1–14) indicated a 17 % positive change in the mean scores increasing from 4.26 to 4.61 (scale 1–5).

The Australian Unity Wellbeing Index (questions 30–38) effect size was 9 % (scale 0–10), as the mean response of staff increased after the participation in the program from 8.25 to 8.66. Staff scored satisfaction with their Health, Standard of living, Future security, Relationships, Achievements in life, Community, Spirituality and Life since the intervention. The most satisfactory post-interventional response was provided on Future security which was estimated even higher than the national benchmark (Cummins and Schafer 2011). The lowest satisfaction post-interventional score was provided on Health.

The 5 % effect size for the workforce engagement survey (questions 40 to 54) indicated that participants experienced minor changes. The 1 % effect size for the K10 (questions 19 to 23) was small and not statistically significant indicating that participants experienced no significant changes with psychological distress after participation in the workshop (Table 2).

## Discussion

This study assessed a three month post-intervention questionnaire of the empowerment program delivered to Indigenous child protection staff. Measuring empowerment, wellbeing and workplace engagement amongst child protection agency staff provides an understanding of how participants coped with stress and demands on their time, as well as perceptions of personal accomplishment and overall satisfaction with life. The assessment provided an opportunity to develop a quantitative framework that captures the social emotional wellbeing outcomes of the empowerment intervention among Indigenous people.

This study found that the sensitivity of surveys to detect changes in emotional development varied. Pre-and post-surveys’ exploratory analysis revealed that the GEM survey, ‘a topic-specific instrument’, which was developed to measure change in dimensions of empowerment as defined and described by Aboriginal Australians who participated in the FWB programme, was the most sensitive demonstrating a 17 % positive change. The Australian Unity Wellbeing Index as a general empowerment instrument showed less sensitivity with a 9 % positive change. The least sensitive measures were the worker engagement survey and K10 with 5 % and 1 % effect sizes respectively.

The study outcome supports findings by Rissel et al. (1996) that a topic specific empowerment instrument, in our case the GEM survey, demonstrated better predictive validity than general empowerment surveys such as for example K10. K10 was developed as a clinical measurement tool to detect non-specific anxiety or depression or psychological distress experienced in the four weeks prior to screening (Kessler et al. 2003). K10 may not be appropriate as a measure for the empowerment interventions in the particular setting.

The findings may have stronger measurable impacts when offered to more vulnerable groups, such as Indigenous participants seeking to improve employment prospects, educational outcomes, conflict resolution skills and ultimately community social cohesion. The finding of 17 % effect size in a more socio-economically stable study sample may be an indication that this effect size is an underestimate of the true impact should this empowerment intervention be offered and measured in a group of people in greater need. Awareness of the social and emotional aspects of life as well as higher levels of empowerment are likely to be enhanced among this sample, compared with Indigenous people who are not engaged in wellbeing activities or work. Different response characteristics, reliability scores and psychometric properties may emerge in other groups and settings. More work is required to explore and test measures across different settings. The study sample represented a population of a remote-dwelling Indigenous workforce. Hence results of the current study are likely to be relevant to other workforce development empowerment interventions among remote Indigenous workers.

### Limitations

The characteristic of the sample consisting of employed participants makes the sample unique and can be considered as a limitation. Despite the lack of randomisation, the pre/post design was implemented in real life service delivery context; therefore the methodology of the current study is feasible and acceptable to service providers. Another limitation to this study was the inability to link pre and post survey responses, which was due to de-identified data collection methods. This was only realised in hind-sight and meant that data could not be ‘matched’ in subsequent analysis. If matched data had been obtained, a smaller sample size may have been adequate, as the matched analysis would providing tighter confidence limits. Future studies in this area should ensure their study design adequately identifies subjects to enable data – matching.

## Conclusion

This study extended qualitative research and identified the best measurement tool for detecting outcomes of empowerment programs. The research recorded a 17 % effect size in the sample of child protection agency staff. The GEM was found the most sensitive and the most tangible measure that captures improvements in communication, conflict resolution, decision making and life skill development. The GEM and Australian Unity Wellbeing Index could be recommended as routine measures for empowerment programs assessment among similar remote area workforce.

The findings of the current study may have stronger measurable impacts when offered to more vulnerable groups, such as Indigenous participants seeking to improve employment prospects and community social cohesion.

## Abbreviations

FWB: Family Wellbeing
GEM: Growth and Empowerment Measure
K10: Kessler psychological distress scale 10
